# Willingness to Participate in HIV Therapeutic Vaccine Trials among HIV-Infected Patients on ART in China

**DOI:** 10.1371/journal.pone.0111321

**Published:** 2014-11-05

**Authors:** Yuan Dong, Xiaoxing Shen, Ruizhang Guo, Baochi Liu, Lingyan Zhu, Jing Wang, Linxia Zhang, Jun Sun, Xiaoyan Zhang, Jianqing Xu

**Affiliations:** 1 Shanghai Public Health Clinical Center and Institutes of Biomedical Sciences, Key Laboratory of Medical Molecular Virology of Ministry of Education/Health, Shanghai Medical College, Fudan University, Shanghai, China; 2 Bloomberg School of Public Health, John Hopkins University, Baltimore, Maryland, United States of America; 3 State Key Laboratory for Infectious Disease Prevention and Control, China CDC, Beijing, China; University of Rochester, United States of America

## Abstract

**Background:**

More and more HIV therapeutic vaccines will enter clinical trials; however, little is known about the willingness to participate (WTP) in HIV therapeutic vaccine trials among HIV-positive individuals.

**Objective:**

To investigate the WTP in HIV therapeutic vaccine trials among Chinese HIV-infected patients.

**Methods:**

We conducted a cross-sectional survey on HIV-positive inpatients and outpatients at Shanghai Public Health Center. A total of 447 participants were recruited into this study. Following an introduction with general information on HIV therapeutic vaccine and its potential effectiveness and side effects, each participant completed a questionnaire in a self-administered form. The questionnaires covered demographics, high-risk behaviors, clinical characteristics and willingness to participate in HIV therapeutic vaccine trial.

**Results:**

The overall willingness to participate in HIV therapeutic vaccine trials was 91.5%. Interestingly, multivariate logistic regression analyses demonstrated that the willingness was higher for those sexually infected by HIV (odds ratio [OR]: 4.36; 95% confidence interval [CI]: 1.53–12.41), diagnosed as HIV-1 infection for greater than 5 years (OR: 7.12, 95% CI: 1.83–27.76), and with the presence of infectious complications (OR: 2.75; 95% CI: 1.02–7.45). The primary reason for participation was to delay or reduce antiretroviral treatment (ART) and to avoid ART side effects (76.6%), and then followed by delaying disease progression (74.9%), increasing immune response to suppress opportunistic infections (57.7%) and preventing the development of drug resistance (37.1%). Reasons for unwillingness to participate mainly included concern for safety (37.0%), lack of knowledge on therapeutic vaccine (33.3%), and satisfaction with ART effectiveness (22.2%).

**Conclusions:**

The WTP in HIV therapeutic vaccine trials was high among HIV-infected Chinese patients. HIV^+^ subjects who acquired infection through sexual contact and who were diagnosed for more than 5 years may represent a good candidate population for enrollment in therapeutic vaccine trials.

## Introduction

According to the UNAIDS Global Report 2013, an estimated 35.3 million people are living with HIV worldwide, with 2.3 million of those people newly infected in 2013 [1]. It is estimated that about 780,000 people are currently living with HIV/AIDS in China, with 434,000 cases reported to the National Health and Family Planning Commission of China until October 2013 (http://www.gov.cn/jrzg/2013-12/01/content_2539529.htm). The strategies for controlling the global HIV epidemic include escalating the usage of condoms, developing vaccine and vaginal/rectal microbicides and implementing treatment as prevention [2], [3]. As an effective vaccine remains as the most cost-effective approach and less depends on human behavior for success [2], thus, the development of an effective and safe vaccine for prevention of AIDS remains as a global public health priority and the greatest opportunity to eventually end the AIDS pandemic [2], [4], [5]. However, after thirty years of research, it remains elusive whether a protective vaccine will be developed, despite strong optimism from previous trials [6], [7]. Thus far, antiretroviral treatment (ART) is the only effective tool for treating persistent HIV-1 infection, which effectively contains HIV replication and delays HIV-related morbidity and mortality [8]. Nevertheless, ART was only accessible to 9.7 million people worldwide at the end of 2012, mainly due to the high cost of ART [1]. In addition, the need for life-long strict adherence to ART regimens, the potential ART side effects and the emergence of multi-drug-resistant viruses pose great challenges to ART treatment [9].

Therapeutic vaccination could be very useful in several ways. First, therapeutic vaccines raise immune responses that target sequence sites that are different from the ART drug targets [10]–[14]. Therefore, therapeutic vaccination could synergize with and strengthen the ART regimen, which would enhance the inhibitory effect on HIV replication, thus improving ART efficacy and reducing the development of drug resistance [9]. Second, therapeutic immunization could be used for “shock and kill” therapy. In combination with ART, the vaccine-stimulated CD8^+^ T cells could cause cytotoxicity of pharmacologically reactivated latent HIV-infected cells and help purge the HIV latent reservoir [15]. Currently, however, no therapeutic vaccine against HIV has been licensed and marketed. Only one therapeutic HIV vaccine, designated as Remune, has completed a Phase III trial, which enrolled 2527 participants at 77 centers in the United States (Immune Response Corp [IRC], Carlsbad, CA) [16]. However, this trial was unsuccessful at improving clinical disease outcome and reducing overall mortality. Currently, a number of HIV-specific therapeutic vaccines, including inactivated HIV viruses, plasmid DNA encoding HIV antigens and recombinant viral vectored HIV vaccines are currently being tested [9]. In addition, autologous dendritic cells either loaded with HIV immunogens or transfected with mRNA encoding HIV antigens are being employed as therapeutic vaccination tools [17].

Considering that an HIV therapeutic vaccine could potentially enhance immune responses and, therefore, delay disease progression and/or reduce the burden of ART, the HIV research community in China is increasing its effort to develop HIV therapeutic vaccines. In fact, one such vaccine has been approved for a Phase I clinical trial (personal communication), and several others, including one from our own lab, are currently moving towards clinical trials. Therefore, it is urgent to gain information regarding the HIV-positive population’s willingness to participate (WTP) in such research studies. In particular, the factors influencing the WTP in and the expectations from HIV-1 therapeutic vaccine trials should be defined because they are critical in preparing the therapeutic vaccine clinical trials and for motivating HIV-positive individuals to engage in those future clinical trials. WTP in preventive HIV vaccine trials has been widely investigated in several countries [18]–[22]; however, only limited information on willingness to participate in HIV therapeutic vaccine trials is available [23]. To gain knowledge on this aspect, we carried out the present study to assess the WTP and its predictors among HIV-infected individuals.

## Methods

### Ethics Statement

Written informed consents were obtained from the participants. The study was reviewed and approved by the Ethics Committee of Shanghai Public Health Clinical Center.

### Subjects and Data Collection

This cross-sectional study was conducted among HIV-infected inpatients and outpatients from the Shanghai Public Health Clinical Center (SPHCC). Currently, SPHCC is the only Shanghai municipal government-authorized hospital providing treatment to HIV/AIDS patients. SPHCC has 50 beds for inpatient treatment of AIDS patients with severe illnesses and routinely provides ART to more than 3000 outpatients, including 400 HIV and TB co-infected patients. Patients are not only from the Shanghai urban and rural areas but also from the Anhui, Jiangsu and Zhejiang Provinces. The study was a self-administered survey. Our study participants were enrolled from 2 resources, including HIV positive outpatients above the age of 18 who visited the SPHCC for routine ART medicine and are willing to complete the self-administered survey; and HIV positive inpatients at SPHCC above the age of 18 and are willing to complete the self-administered survey. All patients were on ART. Our goal was to reach 450 HIV-infected subjects as this sample size is likely to be sufficient for a Phase II clinical trial of a therapeutic vaccine. In total, 500 HIV-positive subjects were approached and 451 agreed to participate in our survey, therefore, about 10% (49 subjects) of the HIV-positive patients rejected to participate in our survey. The major reason for not participating in this survey was privacy concerns. After the exclusion of 4 subjects who did not offer their WTP, 447 subjects remained for information collection and analysis. After informed consent was obtained, each participant was given a pen, a questionnaire and an introduction to an HIV therapeutic vaccine and its potential effectiveness and side effects. The following information was prepared in conjunction with vaccine developer and provided to the participants: “Although scientists are working hard to develop HIV therapeutic vaccines, none have been marketed. Therapeutic vaccines could potentially raise host immunity to fight against HIV by using a different mechanism from ART. Therapeutic vaccination could be integrated with an ART regimen to potentially enhance the therapeutic efficacy or reduce the burden of ART. Chinese scientists are also developing therapeutic vaccines and have shown that these vaccines are capable of stimulating vigorous immune responses. However, it remains unknown whether these vaccines will work in humans; thus, these vaccines need to be tested in clinical trials. Therapeutic vaccination poses no risk for HIV infection, but it may cause discomfort and mild pain at the injection site. Although unexpected, severe side effects may occur. Clinicians will closely monitor your condition during the clinical trial.” Following this introduction, structured questionnaires were completed by all participants.

Data were collected through the questionnaires in the first visit. Closed ended questions with one possible answer were listed as following: demographic characteristics (age, gender, ethnicity, residence, education, annual income, marital status), behavioral characteristics (routes of HIV infection, sexual preference, the number of lifetime sexual partners), clinical characteristics (CD4 counts, years since HIV-1 diagnosis), and willingness to participate (a yes or no response to the description of therapeutic vaccines). Three partially closed questions with more possible answers were also included: chronic medical diseases (cardiovascular disease, such as hypertension and/or heart disease; diabetes; HBV infection; HCV infection; etc.), infectious complications (pneumonia, herpes zoster, tuberculosis, etc.), non-infectious complications (idiopathic thrombocytopenic purpura, B cell lymphoma, cervical cancer, etc.). In a subsequent visit we determined their main motivations and main concerns. Regarding the motivations for participating, subjects willing to participate were able to select one to three out of the seven closed answer choices, including: to delay or reduce ART and thereby to avoid ART side effects[24]–[26], to delay disease progression [27], [28], to increase immune response to suppress opportunistic infections [28]–[31], to prevent drug resistance [32], to earn monetary reimbursement [33], to reduce potential transmission[31], [34]–[36] and to be encouraged by family support [18], [19]. Participants who were not willing to participate were asked to answer the open-ended question “What is the main reason for your decision?” All information was analyzed in the association with the willingness to participate in hypothetical HIV therapeutic vaccine trials.

### Data analyses

Univariate logistic regression analysis was performed to evaluate the associations of WTP with demographic, behavioral and clinical characteristics. Variables that were significant at a level of *P*<0.1 were fitted in a multivariate logistic regression model with a backward, stepwise approach, and only the factors with a significance level of 0.05 were reported. Data were analyzed using SPSS (version 17.0) and Stata (version 12.0). Stata 12.0 was used to perform exact logistic regression for those categories having very low frequencies (Table 1: ethnicity; Table 2: chronic medical diseases of HBV and others, infectious complications of pneumonia and herpes zoster, and non-infectious complications).

**Table 1:** Association between sociodemographic/behavioral characteristics and willingness to participate (WTP) in HIV therapeutic vaccine trials.

		Intention to accept vaccination			
Variable	Total	Yes	No	OR (95% CI)	*P* value
**Age**					0.78
<30	82 (18.5)	75 (91.5)	7 (8.5)	1.00	
30–49	244 (55.0)	222 (91.0)	22 (9.0)	0.94 (0.39–2.29)	0.90
>50	118 (26.6)	109 (92.4)	9 (7.6)	1.13 (0.40–3.17)	0.82
**Gender**					0.94
Men	398 (89.8)	364 (91.5)	34 (8.5)	1.00	
Women	45 (10.2)	41 (91.1)	4 (8.9)	0.96 (0.32–2.83)	0.94
**Ethnicity** a					1.00
Han	438 (99.5)	400 (91.3)	38 (8.7)	1.00	
others	2 (0.5)	2 (100.0)	0 (0.0)	4.39 (0.00–56.88)	1.00
**Residence**					0.67
Village	81 (18.3)	75 (92.6)	6 (7.4)	1.00	
City	361 (81.7)	329 (91.1)	32 (8.9)	0.82 (0.33–2.04)	0.67
**Marital status**					0.27
Unmarried	197 (44.8)	182 (92.4)	15 (7.6)	1.00	
Married	212 (48.2)	195 (92.0)	17 (8.0)	0.95 (0.46–1.95)	0.88
Divorce	31 (7.0)	26 (83.9)	5 (16.1)	0.43 (0.14–1.28)	0.13
**Education**					0.67
Junior high school and lower	108 (24.4)	97 (89.8)	11 (10.2)	1.00	
Senior high school and technical secondary school	135 (30.5)	125 (92.6)	10 (7.4)	1.42 (0.58–3.47)	0.45
Junior college	90 (20.4)	83 (92.2)	7 (7.8)	1.35 (0.50–3.63)	0.56
Undergraduate and above	109 (24.7)	100 (91.7)	9 (8.3)	1.26 (0.50–3.18)	0.62
**Annual income (RMB)**					0.57
<20000	148 (34.6)	131 (88.5)	17 (11.5)	1.00	
20000–40000	152 (35.5)	142 (93.4)	10 (6.6)	1.84 (0.82–4.17)	0.14
40000–100000	95 (22.2)	88 (92.6)	7 (7.4)	1.63 (0.65–4.10)	0.30
>100000	33 (7.7)	29 (87.9)	4 (12.1)	0.94 (0.30–3.00)	0.92
**Routes of HIV infection**					0.18
Blood transfusion	80 (19.4)	73 (91.3)	7 (8.8)	1.00	
Drug abuse	11 (2.7)	7 (63.6)	4 (36.4)	0.17 (0.04–0.72)	**0.02**
Sexual transmission	322 (78.0)	303 (94.1)	19 (5.9)	1.53 (0.62–3.77)	0.36
**Sexual preference**					0.14
Heterosexual	195 (47.6)	173 (88.7)	22 (11.3)	1.00	
Homosexual	189 (46.1)	180 (95.2)	9 (4.8)	2.54 (1.14–5.68)	**0.02**
Bisexual	26 (6.3)	23 (88.5)	3 (11.5)	0.98 (0.27–3.52)	0.97
**Number of lifetime sexual partners**					0.18
<2	111 (28.2)	98 (88.3)	13 (11.7)	1.00	
≥2	283 (71.8)	262 (92.6)	21 (7.4)	1.66 (0.80–3.43)	0.18

a the variable was analyzed with exact logistic regression.

**Table 2:** Association between clinical characteristics and willingness to participate (WTP) in HIV therapeutic vaccine trials.

		Intention to accept vaccination			
Variable	Total	Yes	No	OR (95% CI)	*P* value
**CD4 count (cells/µl)**					0.49
<200	125 (29.7)	111 (88.8)	14 (11.2)	1.00	
200–400	240 (57.0)	223 (92.9)	17 (7.1)	1.65 (0.79–3.48)	0.18
>400	56 (13.3)	52 (92.9)	4 (7.1)	1.64 (0.52–5.23)	0.40
**Years since HIV diagnosis**					**<0.01**
<5	292 (66.4)	259 (88.7)	33 (11.3)	1.00	
≥5	148 (33.6)	143 (96.6)	5 (3.4)	3.64 (1.39–9.54)	**<0.01**
**Chronic medical diseases**					0.68
No	303 (68.1)	276 (91.1)	27 (8.9)	1.00	
Yes (total)^a^	142 (31.9)	131 (92.3)	11 (7.7)	1.17 (0.56–2.42)	0.68
Cardiovascular disease	72^h^	67 (93.1)	5 (6.9)	1.31 (0.49–3.53)	0.59
Diabetes	64^i^	61 (95.3)	3 (4.7)	1.99 (0.59–6.77)	0.27
HBV infection^b^	17^j^	16 (94.1)	1 (5.9)	1.56 (0.23–68.04)	1.00
HCV infection	75^k^	72 (96.0)	3 (4.0)	2.35 (0.69–7.96)	0.17
Others^c^	25^l^	24 (96.0)	1 (4.0)	2.34 (0.35–100.1)	0.69
**Infectious complications**					**0.02**
No	253 (57.2)	224 (88.5)	29 (11.5)	1.00	
Yes (total)^d^	189 (42.8)	180 (95.2)	9 (4.8)	2.59 (1.20–5.61)	**0.02**
Pneumonia^e^	80^m^	78 (97.5)	2 (2.5)	2.24 (1.11–6.67)	**0.02**
Herpes zoster^f^	115^n^	113 (98.3)	2 (1.7)	1.94 (1.21–4.00)	**<0.01**
Others	54^o^	47 (87.0)	7 (13.0)	0.87 (0.36–2.10)	0.76
**Non-infectious complications** g					0.51
No	426 (96.6)	388 (91.1)	38 (8.9)	1.00	
Yes	15 (3.4)	15 (100.0)	0 (0.0)	2.05 (0.34–)	0.51

b,c,e,f,g these variables were analyzed with exact logistic regression.

a,d logistic regression analysis were complicated through several steps, as non-infectious group = reference group, infectious group was considered as one group: Yes (total) or each disease was separately put into the model.

g non-infectious complications included: idiopathic thrombocytopenic purpura, B cell lymphoma, cervical cancer, etc.

h Cardiovascular disease were hypertension and/or heart diseases. 72 cases included 38 infected with HCV and 6 had diabetes or HBV or other diseases.

i 64 cases included 49 infected with HCV and 5 had cardiovascular disease or HBV or other diseases.

j 17 cases included 3 had cardiovascular disease or diabetes or HCV.

k 75 cases included 59 had diabetes, 4 had cardiovascular disease.

l others included: nephritis, gastritis, hyperlipidemia, gout, etc.

m 80 cases included 51 co-infected with herpes zoster, 6 co-infected with other diseases.

n 115 cases included 51 co-infected with pneumonia, 7 co-infected with other diseases.

o others included : tuberculosis, cryptococcal meningitis, toxoplasmic encephalitis, cytomegalovirus/fungal infection, syphilis, Kaposis sarcoma, etc.

## Results

### Demographic, behavioral and clinical characteristics

Between June and October 2012, 451 HIV-positive individuals consented to participate in this study. After excluding 4 subjects for failure to provide WTP data, a total of 447 subjects completed the study. Overall, 409 subjects (91.5%) were “willing to participate”, and 38 subjects (8.5%) were “not willing to participate”. Among the 447 participants, the majority were men (89.8%), of the Han ethnicity (99.5%) and 30 to 49 years old (55.0%). Among all demographic and behavioral variables, homosexuality as a sexual preference and drug abuse as an infection route for HIV were the only factors associated with WTP with a significance level less than 0.1 (Table 1). Because only 11 participants reported HIV infection due to drug abuse, it is possible that such a small sample size may cause bias. Three factors, having divorced, having an annual income at 20000–40000 RMB (Chinese currency, 1 dollar≈6.35 Yuans in RMB in 2012) and having more than 2 sexual partners, had their *P* values close to 0.10, whereas all other variables had much higher *P* values (Table 1).

Five categories of clinical characteristics, CD4 count, years since HIV diagnosis, presence of chronic medical disease, infectious complications and non-infectious complications, were included in Table 2. Among these clinical characteristics, years since HIV diagnosis and infectious complications (specifically, pneumonia and herpes zoster) were associated with WTP with a significance level below 0.10, whereas CD4 count, presence of chronic medical diseases and non-infectious complications had *P* values >0.10 (Table 2).

### Factors associated with WTP

Multivariate logistic regression analyses revealed that three variables independently predicted the willingness to participate in HIV therapeutic vaccine clinical trials (Table 3). These included sexual transmission of HIV (OR: 4.36; 95% CI: 1.53–12.41; *P*<0.01, compared with blood transfusion), an HIV diagnosis time of more than 5 years (OR: 7.12, 95% CI: 1.83–27.76, *P*<0.01, compared with diagnosed less than 5 years) and the presence of infectious complications (OR: 2.75, 95%CI: 1.02–7.45, *P*<0.05, compared with those without infectious complications).

**Table 3:** Multivariate logistic regression analysis of factors associated with willingness to participate (WTP) in HIV therapeutic vaccine trails.

	Intention to accept vaccination	
Variable	OR (95% CI)	*P* value
**Routes of HIV infection**		
Blood transfusion	1.00	
Drug abuse	0.27 (0.05–1.46)	0.13
Sexual transmission	4.36 (1.53–12.41)	**<0.01**
**Years since HIV diagnosis**		
<5	1.00	
≥5	7.12 (1.83–27.76)	**<0.01**
**Infectious complications**		
No	1.00	
Yes	2.75 (1.02–7.45)	**<0.05**

### Reasons associated with WTP and non-WTP

Of the 409 subjects who responded as willing to participate in a therapeutic vaccine trial, 175 (42.8%) provided their reasons in a subsequent visit (Table 4). The primary motivation for WTP in HIV therapeutic vaccine trials was to delay or reduce ART and thereby to avoid ART side effects (76.6%), then followed by delaying disease progression (74.9%), increasing immune response to suppress opportunistic infections (57.7%), preventing drug resistance (37.1%), reducing the potential transmission (36.6%), earning economic reimbursement (15.4%), and being encouraged by family support for participation (5.1%).

**Table 4:** Reasons for participating or not participating in HIV therapeutic vaccine trails.

Willing?	Reason	Number of respondents	Percentage (%)
Yes(n = 175)^a^	Delay or reduce ART, avoid ART side effects	134	76.6
	Delay disease progression	131	74.9
	Increase immune response to suppress opportunistic infections	101	57.7
	Prevent drug resistance	65	37.1
	economic incentives	27	15.4
	Reduce potential transmission	64	36.6
	Family support	9	5.1
No (n = 27)^b^	Concern about the safety of the therapeutic vaccine	10	37.0
	Lack of knowledge on therapeutic vaccine	9	33.3
	Satisfaction with ART effectiveness	6	22.2
	Too old	1	3.7
	Too busy	1	3.7

a Of the 409 subjects who responded as willing to participate, 175 provided their reasons.

b Of the 38 subjects who responded as not willing to participate, 27 provided their reasons.

Of the 38 subjects who responded as not willing to participate in a therapeutic vaccine trial, 4 subjects indicated that they needed further consideration, but they did not provide detailed information. Twenty-seven subjects (71.1%) also indicated that they needed further consideration but they provided their reasons. Of the 27 subjects who provided their reasons, 10 subjects (37.0%) indicated that they were concerned about the safety of the vaccine, and 9 subjects (33.3%) indicated that they did not have sufficient knowledge about the therapeutic vaccine but would like to pay attention to its progress. The remaining 8 subjects (29.6%) were not interested in participating in a therapeutic vaccine trial because they were either satisfied with ART (6/8), considered themselves too old (1/8), or were too busy for a trial (1/8).

## Discussion

As an important therapeutic strategy, a therapeutic vaccine will likely be integrated into ART regimens to enhance the efficacy of treatment or potentially to eradicate HIV infection in the future [32]. However, only very limited information on the WTP in HIV therapeutic vaccine trials is currently available [23]. Asia is the second most severely affected region, after Sub-Saharan Africa, and may greatly benefit from an effective therapeutic vaccine [37]. Therefore, understanding the WTP in therapeutic vaccine trials among this population will be crucial for the preparation and the success of therapeutic vaccine clinical trials. Accordingly, the questions in our study questionnaire were specifically designed to determine factors that might motivate or impede HIV-infected individuals to approach to therapeutic vaccine trial.

WTP in HIV preventive vaccine trials has been widely investigated, which has enlightened the recruitment of volunteers for clinical trials. Studies reported a wide range of HIV preventive vaccine acceptability, ranging from less than 40% to over 90% [18]–[22], [33], [38]–[43], and noted several motivators and barriers for HIV vaccine trial participation. Being homosexual, of an older age, aware of HIV infection risk, aware of HIV vaccine, free insurance and/or medical care and monetary incentives are motivators, whereas the fear of vaccine-induced HIV infection, being African American (in comparison to white), uncertainty about vaccine efficacy, side effects/safety concerns, mistrust of government or research institutes and associated stigma and discrimination are barriers [20], [33], [38], [40]–[43]. There were several studies on WTP in HIV preventive vaccine trials conducted in different areas of China. In northwest China (Urumqi City), WTP in HIV preventive vaccine trials among IDUs was 92.0% (74.3% definitely willing and 17.7% most likely willing). WTP was positively associated with a history of sex with a drug-using partner, needle and syringe sharing with a new drug use partner in the past 3 months, perceived family support for participation, and perceived vaccine protection against HIV infection; alternatively, WTP was negatively associated with the perceived risk of social stigma and isolation for participation [19]. In Beijing, WTP of MSM was 70.9% (35.8% definitely willing and 35.1% most likely willing), and the analysis suggested that perceived family support, perceived protection against HIV infection and fear that participation would result in social distancing were associated with WTP [18]. In northeast China (Shenyang, Fushun and Anshan), WTP was 76.7% among MSM, and it was positively associated with spouses and family protection, family support and economic incentives [44]. In our study, we also included sociodemographic variables (age, gender, ethnicity, education, annual income and marital status) and high risk factors (sexual preference and the number of lifetime sexual partners) in our questionnaire study. Consistent with previous studies, our current study revealed that age, gender, education, marital status and the number of sex partners have no correlation with WTP [20], and homosexuals are more willing to participate in therapeutic vaccine trials than those reported as heterosexuals [42].

However, because participants for therapeutic vaccine trials are different from preventive vaccine trials, the factors influencing WTP may be very different. Participants for preventive vaccine trials are high risk populations [18], [19], [42] or general population [45], [46]; whereas participants in therapeutic vaccine trials are HIV infected subjects [23]. Thus in this study, we designed questions to encompass clinical characteristics of participants including: routes of HIV infection, CD4 counts, years since HIV diagnosis, chronic medical diseases, infectious complications and non-infectious complications.

A study on the WTP in HIV therapeutic vaccine trials among IDUs observed a 54% acceptance rate [23], which determined the role of three cognitive factors: HIV treatment optimism, self-efficacy beliefs, and knowledge of vaccine trial concepts in relation to WTP in a hypothetical phase 3 therapeutic HIV vaccine trial, and observed that an increase in self-efficacy had a statistically significant positive association with WTP. Differently, as our study was designed to identify sociodemographic, infection routes and clinical characters associated with WTP to facilitate the recruitment of volunteers for clinical trials in future, we also enrolled HIV^+^ patients infected through sexual contact and blood transfusion in addition to IDUs, and observed that 91.5% of HIV-positive participants are willing to participate in a hypothetical HIV therapeutic vaccine trial, and the high WTP was mainly attributed to the very high acceptance of vaccine trial in patients infected by sexual contact. Similar to previous observation, only 63.6% HIV-infected IDUs were willing to receive HIV therapeutic vaccine. Overall, HIV infection route is one of crucial factors influencing WTP. As our questionnaire was developed in conjunction with vaccine developer and the same information will be presented to participants in the enrollment of real therapeutic vaccine trials, we expect that a similar high acceptance is likely to be achieved.

Notably, our study showed several new predictors and barriers. We found that participants infected with HIV through sexual transmission have higher WTP than those infected through blood transfusion or drug use. Thus, individuals who were infected with HIV through sexual behaviors might be good candidates for therapeutic vaccine trials. In addition, our data did show a trend that WTP increased with years since HIV diagnosis, and participants who were diagnosed with HIV for more than 5 years had significantly higher WTP than those diagnosed for less than 5 years, and patients with infectious complications are more willing to participate in therapeutic vaccine trials. These may be due to ART-related toxicity, or agony caused by complications associated with HIV, etc. Years since HIV diagnosis and infectious complications were independent factors that predict the willingness to participate in HIV therapeutic vaccine clinical trials. In accordance with the preferential factors for WTP, to delay or reduce ART to avoid ART side effects and to delay disease progression were reported by more than 70% of individuals, and over 50% reported to increase immune response to suppress opportunistic infections as their motivations to participate in therapeutic vaccine trials. These results indicate that the benefits for participation in HIV therapeutic vaccine trials should be well introduced to participants before the initiation of recruitment in the future clinical trials.

Three reasons are associated with non-WTP, including concern about the safety of the vaccine, the lack of knowledge on the therapeutic vaccine, and the satisfaction with current ART therapy. As the first two accounted for two-thirds of the non-WTP subjects, information on the HIV therapeutic vaccine to improve knowledge and reduce safety concerns is likely to further increase the willingness of HIV-infected subjects to participate in HIV therapeutic vaccine trials. In addition, as family plays a very important role in Chinese traditional culture, it is possible that support from family members will encourage subjects who need further consideration to participate and will help reduce their concern about safety.

Several limitations in our study should be noticed. First, we did not include psychological factors into our study. Altruism and self-efficacy beliefs have been proved as important motivators to participate in an HIV therapeutic vaccine trial of Remune and ALVAC, or in hypothesized Phase III therapeutic HIV vaccine trials [23], [47], which might help us to explain why clinical and demographic variables in our study were related to WTP. Second, other demographic characteristics such as children should be included as it may facilitate patients to approach better treatment under the stimulation of altruism; Third, the WTP could have been considered as continuous variable and the sample size could be expanded to further reduce confidence interval and stabilize the WTP of different factors; Finally, the questions should be worded consistent with Prospect Theory predictions in future [48].

## Conclusions

In conclusion, our study showed that the majority of HIV-infected subjects in the Shanghai area of China are willing to participate in HIV therapeutic vaccine trials. This is true especially for individuals who were infected with HIV through sexual contact, who have been diagnosed for more than 5 years or who have complications from infectious diseases. Introducing the potential benefits and improving the knowledge on the HIV therapeutic vaccines and reducing concerns about vaccine safety are likely to further increase the willingness of HIV-infected subjects to participate in HIV therapeutic vaccine trials.
